# Glomerular Endothelial Cell Stress and Cross-Talk With Podocytes in Early Diabetic Kidney Disease

**DOI:** 10.3389/fmed.2018.00076

**Published:** 2018-03-23

**Authors:** Ilse Sofia Daehn

**Affiliations:** Division of Nephrology, Department of Medicine, Icahn School of Medicine at Mount Sinai, The Charles Bronfman Institute for Personalized Medicine, New York City, NY, United States

**Keywords:** diabetic kidney disease, glomerulus, cross-talk, podocyte, endothelial cell, reactive oxygen species

## Abstract

Diabetic kidney disease (DKD) is one of the major causes of morbidity and mortality in diabetic patients and also the leading single cause of end-stage renal disease in the United States. A large proportion of diabetic patients develop DKD and others don’t, even with comparable blood glucose levels, indicating a significant genetic component of disease susceptibility. The glomerulus is the primary site of diabetic injury in the kidney, glomerular hypertrophy and podocyte depletion are glomerular hallmarks of progressive DKD, and the degree of podocyte loss correlates with severity of the disease. We know that chronic hyperglycemia contributes to both microvascular and macrovascular complications, as well as podocyte injury. We are beginning to understand the role of glomerular endothelial injury, as well as the involvement of reactive oxygen species and mitochondrial stress, which play a direct role in DKD and in other diabetic complications. There is, however, a gap in our knowledge that links genetic susceptibility to early molecular mechanisms and proteinuria in DKD. Emerging research that explores glomerular cell’s specific responses to diabetes and cell cross-talk will provide mechanistic clues that underlie DKD and provide novel avenues for therapeutic intervention.

## Introduction

Diabetic kidney disease (DKD) is one of the major causes of morbidity and mortality in diabetic patients and also the leading single cause of end-stage renal disease (ESRD) in the United States (1) and now a global epidemic (2–4). There has been little progress in treating DKD since angiotensin-converting enzyme (ACE) inhibitors and angiotensin II receptor blockers (ARBs) were shown to delay progression of nephropathy over 20 years ago (5, 6). The current treatments for DKD are limited to hyperglycemic control, blood pressure control, ACE inhibitors, or renin–angiotensin system (RAS) blockade, which can delay the progression to ESRD; however, the absolute risk of renal and cardiovascular morbidity and mortality remains overwhelmingly high (7). Recent renal outcome trails with agents such as thiazolidinediones, DPP-4 inhibitors, bardoxolone, and sulodexide have been unsuccessful; however, exciting new data from trials with GLP1R agonists and SGLT2 inhibitors show beneficial renal effects in patients with type 2 diabetes mellitus (T2DM) (8, 9). Although these new therapies seem promising, they may not reduce occurrence of ESRD (9). Hence, there is urgency from the community for new effective therapies to halt or reverse disease progression.

The prevalence of ESRD is up to 10 times higher in people with diabetes and with much higher health expenditures compared with those without diabetes (7, 10). The complex pathogenesis of DKD is influenced by genetics and by the environment (11–14). The majority of patients with diabetes do not develop DKD despite similar levels of hyperglycemia (15), and this is also the case with experimental mouse models (16, 17). Initially, patients present with microalbuminuria [urinary albumin excretion rate (UAER) 30–300 mg/24 h], which can progress to macroalbuminuria (UAER ≥ 300 mg/24 h) and eventually ESRD (18). However, there is a large proportion of diabetic patients that have decreased renal function in absence of substantial proteinuria (19), suggesting that other mechanisms contribute to kidney damage adding to the complexity of DKD, as well as highlighting the need for better biomarker predictors of progressive kidney failure in this population. It is thought that this will come from a better understanding of the pathogenesis of DKD.

This review aims to highlight the emerging evidence of intra-glomerular cell cross-talk in DKD, with particular focus on endothelial–podocyte communications, redox, cellular targets, and potential opportunities for discovery of new targets for the prevention and treatment of progressive DKD.

## Hyperglycemia and Glomerular Injury—First Hit

Hyperglycemia is the driving force for diabetic complications, and the glomerulus is the primary site of diabetic injury in the kidney. The early stage of DKD is characterized by glomerular hyperfiltration, hypertrophy, mesangial expansion with altered matrix composition, and thickening of the glomerular basement membrane (BM). Podocyte injury resulting in podocyte foot process effacement (20–22) and depletion is a hallmark of progressive DKD (23–25). Podocytes contribute to glomerular permeability with their exceptionally complex morphology that is the key to their physiological function. The development of proteinuria is associated with marked morphological changes in these cells, and this relationship has been described in patients with diabetes both with microalbuminuria and macroalbuminuria (26, 27). There is a correlation between diabetes effects on podocyte morphology and their pathophysiology, all principal features that clinically manifests as proteinuria. Podocytes are terminally differentiated visceral epithelial cells with a limited capacity to regenerate (21), thus the “podocyte depletion paradigm” correlates closely with the development of proteinuria and progressive glomerulosclerosis, and therefore podocytes have been studied extensively as key targets in the evolution of segmental sclerosis lesions.

Many research groups are actively investigating the pathomechanisms of podocyte injury and depletion in DKD and other glomerular diseases. In DKD, the glomeruli are exposed to various noxious stimuli such as high glucose, uric acid, fatty acids, growth factors, cytokines, and hormones, and all these have been associated with podocyte loss in experimental models of DKD. A handful of potential mechanisms for podocyte depletion in DKD have been suggested and discussed, including the induction epithelial-to-mesenchymal transition (EMT). During EMT, cell–cell and cell–extracellular matrix interactions are altered to release epithelial cells from the host tissue (28). This process involves reorganization of the cytoskeleton to enable migration, and an altered transcriptional program is induced to maintain these cells in a mesenchymal phenotype while losing their hallmark epithelial characteristics (28–30). Diabetes-induced podocyte EMT may be mediated through several molecular mechanisms such as TGF-β/Smad classic pathway, Wnt/β-catenin signaling pathway (31), integrins/integrin-linked kinase signaling pathway (32), mitogen-activated protein kinases (MAPKs) signaling pathway, Jagged/Notch signaling pathway (33), and NF-κB signaling pathway (34). Although it is still debatable, podocyte depletion may result from decreased podocyte adhesion in DKD, which is a potential consequence of EMT. Podocyte transition to a more mesenchymal nature could result in their detachment facilitated by mechanical forces and shear stress derived from filtrate flow through the filtration slits acting on the foot-processes challenging the attachment of podocytes to the GBM (35–37) and consequent impairment of renal filtration.

In DKD podocyte loss through cell death was first demonstrated by Susztak et al. (38), podocytes exposed to high glucose had increased reactive oxygen species (ROS) *via* NADPH oxidase resulting in apoptosis by activation of MAPK and the caspase-3 cascade *in vitro*. An increase in transferase-mediated dUTP nick-end labeling positive podocyte nuclei coincided with onset of hyperglycemia and with onset of albuminuria in both T1DM and T2DM models. Although apoptosis has been described as the major mechanism by which podocytes die in diabetes and progressive glomerular disease by our group and others (39–42), the topic of podocyte cell death, detachment, and loss in glomerular diseases has been discussed extensively elsewhere (43) and remains controversial. Whether the diabetic milieu effects on podocytes could promote podocyte detachment, i.e., preceded by any kind of podocyte cell death, such as apoptosis, necrosis, necroptosis, ferroptosis, podoptosis, and mitotic catastrophe (44–48), still remains under debate and not the focus of this review. The maintenance of the glomerular ultrafiltration barrier requires the interaction of all three glomerular cell types (49, 50) including a multidirectional cross-talk between podocytes, mesangial cells and endothelial cells (51–53), as well as parietal epithelial cells (54, 55). Understanding the key interactions between all the cells in the glomerulus during the development of DKD may provide us with an opportunity for identifying novel interventions that can either stop or reverse its progression.

## Endothelial Dysfunction in DKD

Chronic hyperglycemia contributes to both diabetic microvascular and macrovascular complications of diabetes resulting in thrombotic microaneurysms, which are typical manifestations of endothelial dysfunction in the glomerulus. Glomerular endothelial cells are highly specialized cells with fenestrae and a charged luminal glycocalyx layer (56, 57), which contribute to the filtration barrier (58). Studies have shown that the severity of DKD is correlated with endothelial dysfunction in T1DM and T2DM (59, 60). DKD progression may be tied to increased plasma levels of endothelial cells derived von Willebrand factor, soluble vascular cell adhesion molecule-1, and soluble intercellular cell adhesion molecule-1 in patients (61, 62) and reduction of the endothelial glycocalyx (63, 64). However, the role of endothelial injury in the pathogenesis of DKD remains relatively unexplored.

In DKD-susceptible mouse strains, we have reported changes in the morphology and potential dysfunction of glomerular endocapillaries upon early onset of hyperglycemia by examining the overall glomerular 3D structure with scanning electron microscopy (42). Compared to control non-diabetic glomerular capillary vessels, 3-week diabetic mice showed distinct morphological changes including a significant decrease in number of fenestrae, there were cytoplasmic protrusions, and increased in number of non-fenestrated ridges. Interestingly, podocyte foot-processes widening or effacement were not evident at this time point, suggesting to us that endothelial cell injury precedes podocyte foot-processes effacement and this was an important feature of early DKD with potential consequences in the progression of disease in genetically susceptible mice and potentially in humans.

Endothelial dysfunction could result from hyperglycemia increased ROS, uric acid, and endothelial nitric oxide synthase (eNOS) inactivation, which reduces nitric oxide (NO) levels (65). Endothelial NO plays a critical role in maintaining renal blood flow, regulating glomerular filtration rate, as well as salt and fluid homeostasis; hence, the inactivation of NO particularly through increased oxidative stress has been linked to altered kidney and vascular function [for review, see Ref. (66)]. A vital role for eNOS-derived NO in the pathogenesis of DKD was illustrated in studies comparing diabetic control wild-type and diabetic eNOS knockout (KO) on the C57BL6 DKD resistant background (67). eNOS KO developed overt albuminuria, hypertension, and glomerular mesangiolysis, injured endothelial morphology, thickened glomerular BM, and focal foot process effacement, whereas control mice did not. By correcting changes in eNOS dimerization and phosphorylation, Cheng et al. were able to reduced albuminuria, GBM thickness, and urinary excretion of oxidative stress markers; moreover, it did not affect hyperfiltration or mesangial expansion (68). Along with this observation, diabetic mice with endothelial dysfunction induced by genetic deficiency of eNOS get podocyte injury with heavy albuminuria (69) and maintenance of endothelial levels of the essential eNOS cofactor tetrahydrobiopterin was shown to ameliorate DKD in these mice (70). Finally, the levels of asymmetric dimethylarginin, an endogenous inhibitor of endothelial eNOS, contribute to increased risk of progressive DKD in patients with T1DM (71–73) and eNOS polymorphisms that result in reduced enzyme function have been associated with more advanced diabetic nephropathy (74, 75). Altogether, these reports support the role of endothelial dysfunction in the pathogenesis and progression of DKD.

## The ROS Paradox in DKD

Oxidative stress represents overproduction of ROS relative to antioxidant defenses (76). An imbalance in the oxidation–reduction state has been identified as a major culprit for diabetic complications (77, 78), including DKD (79–81). Increased ROS production triggers renal fibrosis and inflammation and causes significant tissue damage by promoting lipid peroxidation, protein and DNA damage, and mitochondrial dysfunction. In the kidney, ROS are generated by xanthine oxidase, cytochrome P450 systems, uncoupled NO synthase, mitochondrial respiratory chain, and NOXs. Among NOX isoforms, NOX1 has been shown to modulate the p38/p27(Kip1) signaling pathway *via* PKC activation and promotes premature senescence in early stage DKD (82). NOX4- and NOX5-derived ROS are important in glomerular and podocyte injury; however, their regulation and function relative to DKD remains unclear (83). Studies by Babelova et al. (84) demonstrated an increase in albuminuria in diabetic mice with genetic deletion of NOX4, although they reported prominent expression of NOX4 to be in tubular cells. Nox4 was also shown to have anti-apoptotic properties protecting kidneys after ischemia reperfusion injury (85). On the other hand, a study using podocyte-specific inducible NOX4 transgenic mice showed glomerular injury characteristic of DKD (86); hence, the context of NOX4 may be important. You et al. and others (87–89) have shown that NOX1/NOX4 inhibition has renoprotective effects in experimental DKD. More research is needed to understand the roles of NOXs in DKD.

The mitochondria are the main cellular source of energy and of ROS production within cells in response to metabolic demands and/or cellular stress signals (90) and are (among other roles) the central regulators of the intrinsic apoptosis pathway. In fact, abnormalities of glomerular podocyte function linked to mitochondrial disorders are involved in the etiology of glomerular pathology with nephrotic syndrome (91–93). An excessive production of mtROS can damage macromolecules within mitochondria, including lipids, proteins, and mitochondrial DNA (mtDNA) (94), this in turn impairs the synthesis of components of the ETC, as well as reduce the capacity to generate ATP, and potentiate further ROS production (vicious cycle: oxidative stress) resulting in mitochondrial dysfunction (95, 96). mtDNA is a vulnerable oxidation target with a higher mutation rate than nuclear DNA due to vicinity to ETC, a higher rate of replication, lack of protection from histones, and DNA repair mechanisms (96–98). An assessment of metabolites from stage 3 to 4 CKD patients versus controls demonstrated that urinary excretion of citric acid cycle metabolites and of genes regulating these metabolites were reduced in patients with CKD, supporting the emerging view of CKD as a state of mitochondrial dysfunction (99).

Increased mtROS and mitochondrial dysfunction could play a critical role in the pathogenesis of DKD (78). Mitochondrial stress in DKD has been demonstrated to be linked to induction Rho-associated coiled-coil-containing protein kinase1 expression and thus resulting in mitochondria fission by promoting phosphorylation and translocation of dynamin-related protein-1 into the mitochondria (100). This study suggests a critical role for hyperglycemia induced mtROS production, mitochondrial fission, and consequent mitochondrial dysfunction in podocytes and endothelial cells (100). By contrast, superoxide production has been shown to be reduced in the kidneys of a STZ-induced T1DM, and using multiple *in vivo* and *ex vivo* approaches the authors did not observe evidence for enhanced mitochondrial superoxide production in diabetic kidneys. The authors showed evidence of mitochondrial dysfunction, reduced overall mitochondrial content, decreased biogenesis, and increased total urinary ROS (101, 102). This contrasting view to the widely held unifying theory that suggests high glucose drives overproduction of superoxide from mitochondria in diabetic complications, encompasses “mitochondrial hormesis,” supported by the reduction of AMPK, sirtuins, and PGC1α pathways and increased mTOR, reduced mitochondrial ROS, reduced biogenesis, and disease progression (103). This brings up an important consideration, as ROS moieties are essential for specific intracellular signaling pathways that regulate physiological processes and represent normal mitochondrial function. Increased ROS act as second messengers essential in vascular homeostasis (104) and significantly contribute to the immune response (105, 106). Therefore, scavenging all ROS by antioxidants may be dangerous, as we have learned from failures of several large clinical trials using antioxidant therapies on DKD patients (107, 108). With these considerations, Galvan and colleagues developed a two-photon imaging method using a genetically encoded redox biosensor to monitor the dynamic mitochondrial redox state of mice kidneys in real-time. Using this approach, the authors confirmed an increased production of mtROS in the kidneys of diabetic mice (109). As we evolve our understanding of ROS chemistry and cell biology, it becomes clearer that different ROS and their location can have distinct and important roles in the kidney.

Our recently published data is in accordance with mitochondrial-dependent glomerular pathomechanisms in DKD susceptibility (42). We were guided initially by unbiased transcriptomic profiling of glomeruli after onset of diabetes in DKD-susceptible DBA/2J and -resistant C57BL/6J inbred mouse strains. Among differentially expressed transcripts in diabetic DBA/2J mice, genes with well-established functional roles in “oxidative phosphorylation” and “mitochondrial function” were most significantly enriched and antioxidants were downregulated in diabetic DBA/2J susceptible mice, suggesting a redox imbalance in glomeruli. The increase in mitochondria oxidative stress resulted in mtDNA instability and damage, and we discovered that the damage accumulated exclusively in the glomerular endothelial cells, resulting in decreased NO and overall endothelial dysfunction (42), this occurred despite the fact that ROS and metabolic changes may be increased in podocytes (22) as well as other glomerular cells upon chronic exposure to a diabetic milieu. Moreover, prevention of mtROS with a mitochondrial-specific scavenger prevented podocyte loss, albuminuria, and glomerulosclerosis (42). Another study evaluated the mRNA profile of glomeruli and isolated podocytes from diabetic mice kidneys (110). The authors demonstrated distinct upregulated pathways involving mitochondrial function and oxidative stress in the endothelium compartment, in isolated glomeruli. By contrast, isolated podocytes showed changes in the regulation of actin cytoskeleton-related genes as major pathways affected in diabetic mice (110). We showed that increased oxidative damage in glomerular endothelial cells was also detected in human subjects diagnosed with DKD, and oxidized DNA lesion excretion in the urine (8-OHdG) was significantly increased only in patients with progressive DKD (42). In accordance with our data, higher plasma concentrations of 8-OHdG were found to be independently associated with increased risk of progression of kidney disease in T1DM (111). Altogether, there are convincing data showing that oxidative damage is context dependent in DKD and that the glomerular endothelium could be the weakest link under chronic stress, and suggests that podocyte depletion in DKD-susceptible mice could be contingent on endothelial cell mitochondrial dysfunction.

## Second Hit Hypothesis Leading to Proteinuria is Context Dependent

In studying the highly complex nature of diabetes-mediated glomerular cell injury, conflicting results from *in vivo* and/or *in vitro* studies driven hypothesis have led some researchers to propose a “second hit hypothesis” to explain podocyte loss in experimental models of DKD and other glomerular diseases (47, 112, 113). Glomerular cells are tightly intertwined interdependently for proper function, with signal processings that must interpret the environment under normal and stress conditions. Podocytes control endothelial cell growth and survival *via* cross-talk of essential paracrine vascular endothelial growth factor alpha (VEGFA and VEGF-R) (114, 115). Cross-talk also exists between endothelial and mesangial cells (PDGF-B and PDGFR-β) and between podocytes and mesangial cells (CCL21 and CCR7) (116, 117). This bidirectional signal cross-talk enables cells to function effectively. However, in response to changes in the microenvironment (e.g., diabetes), a communication network could provide feedback that may enable cells to tune their signaling activity to organize cytoskeletal dynamics, metabolic output, etc. The molecular mechanisms for glomerular cell cross-talk and feedback regulation in proteinuric glomerular diseases remain poorly understood.

**Figure 1:**
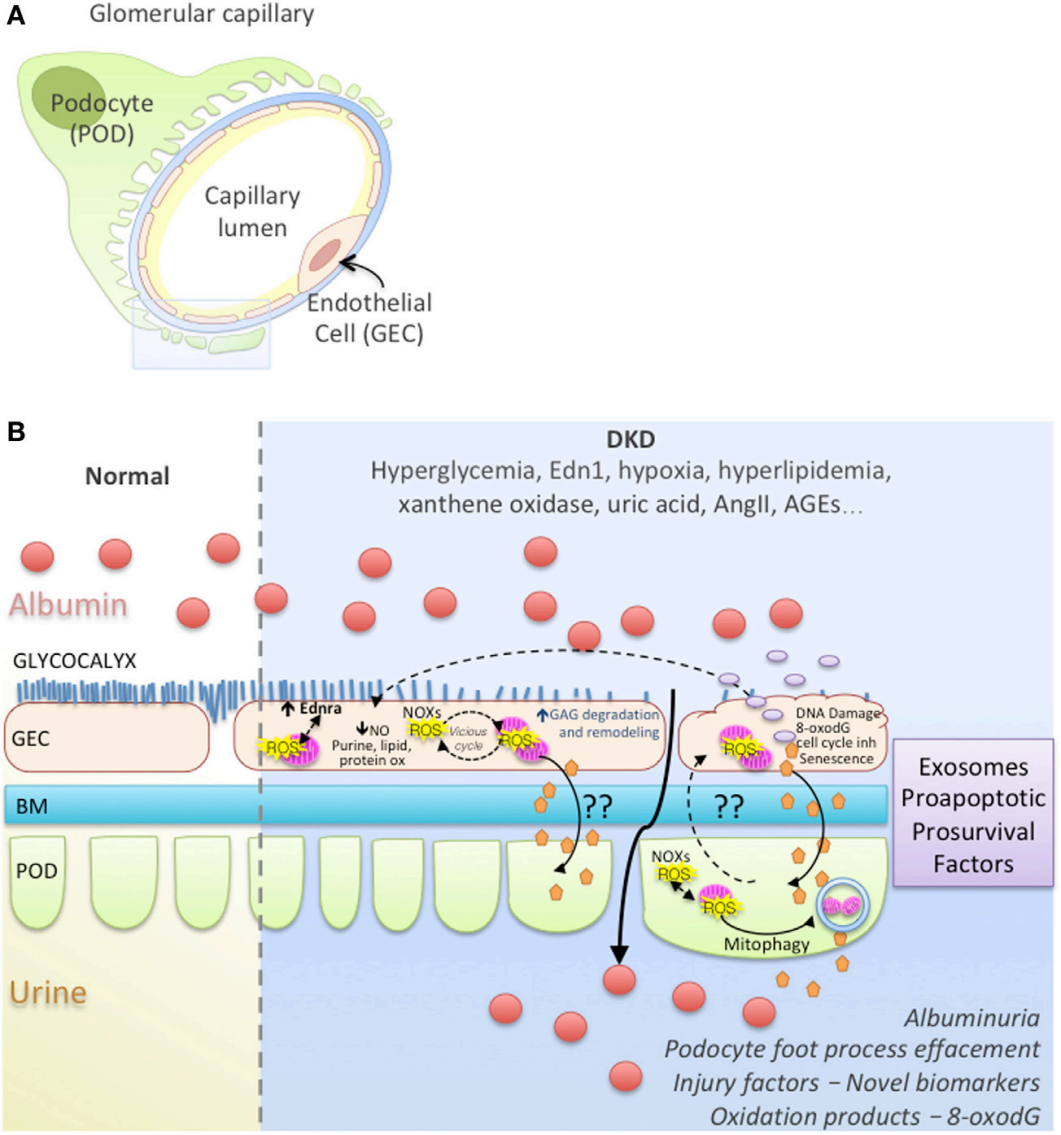
Working model of endothelial cell–podocyte cross-talk in diabetic kidney disease (DKD). **(A)** Glomerular capillary showing a podocyte (POD; Green), basement membrane (BM; blue), endothelial cell (GEC; pink). **(B)** Magnified insert from **(A)** illustrates normal and DKD environment; GEC injury with ROS vicious cycle, mitochondrial dysfunction, glycocalyx loss, endothelial remodeling and cross-talk with podocytes, albuminuria, podocyte foot process effacement in DKD.

In most diseases causing glomerulosclerosis, transforming growth factor β (Tgfb) expression in podocytes is a stress response signal associated with segmental sclerosis and podocyte loss (118–120). In a model of FSGS, podocyte injury initiated by activating Tgfb signaling specifically in podocytes, we showed an increase in the release of endothelin-1 by podocytes, which acted on increased endothelin receptor type A (Ednra) on adjacent glomerular endothelial cells resulted in endothelial cell mitochondrial oxidative stress and endothelial cell dysfunction (121). Surprisingly, glomerular endothelial mitochondrial oxidative stress and dysfunction was absolutely essential for subsequent podocyte loss, illustrating a podocyte-to-endothelial-to-podocyte cross-talk (121). A similar stressed endothelial-to-podocyte cross-talk *via* mitochondrial oxidative stress in endothelial cells downstream from Edn-1/Ednra could also underlie segmental lesions in DKD and highlight a potential mechanism for the proven renoprotective activities of EDNRA inhibitors (42). Work from our laboratory and others have shown that Tgfb signaling in podocytes increases mitochondrial activity (respiration rate) (122, 123). Although Abe et al. (122) reported a concomitant increase in ROS production and mitochondrial membrane potential *via* and activation of mTOR, we did not detect ROS damage in podocytes (123). Podocytes are quite resilient with effective high level of constitutive autophagy (124–126) that could efficiently remove damaged organelles. Perhaps Tgfb signaling activation increases podocytes susceptibility to cross-talk messaging factors from neighboring endothelial cells with sustained injury that might represent a “second hit” for podocytes, leading to depletion and proteinuria *in vivo*. Recently, a study showed that secreted exosomes derived from high glucose-treated endothelial cells could mediate EMT and dysfunction of podocytes in a paracrine manner and activating canonical Wnt/β-catenin signaling (127). Interestingly, podocyte injury in a DKD resistant mice strain was detected when KLF2 expression was decreased specifically in endothelial cells of these mice (128). Figure 1 illustrates hypothetical cross-talk between glomerular endothelial cells and podocytes, ROS, ROS damage, mitochondrial dysfunction in DKD.

## A Need for Disruption in Our Approach to Treating DKD

The need for renal replacement, the mortality risk, and the financial burden are tremendously high among diabetics with kidney disease. Hence, halting or reversing disease progression will significantly impact patients living with this debilitating disease.

New treatments that aim to restore endothelial function could be an effective strategy for treating DKD as shown in a study that targeted cGMP; a key messenger for NO signaling. The study showed reduced progression of renal damage in the ZSF1 rat with diabetic nephropathy in the absence of significant hemodynamic effects (129). This approach is currently been examined clinically. Renoprotection in T2DM has been reported by preservation of mitochondrial function with CoQ10, which acts as both an acceptor of electrons and a ROS scavenger (130). Also, the mitochondria-targeted analog mitoubiquinone (131) has been shown to protect kidneys from diabetic injury by prevention of nuclear accumulation of pro- phospho- Smad2/3 and β-catenin or by Nrf2/PINK1-mediated mitophagy in tubules (132, 133). Preclinical studies with mitochondrial-targeted scavanger Szeto-Schiller peptide (Bendavia™) have demonstrated benefits in rodent models of DKD by improving mitochondrial bioenergetics (134), alleviating proteinuria, urinary 8-OHdG levels, glomerular hypertrophy, and accumulation of renal fibronectin and collagen IV (135). Clinical trials are underway to evaluate the cardiac and renal benefits using this targeted antioxidant mechanism in patients. Although mitochondria are a promising therapeutic target, the question of whether mitochondrial stabilization and mtROS inhibition can improve patient endpoints in large, randomized DKD clinical trials still remains.

We are at an exciting time, where as a community we understand that this multi-dimensional, multi-cellular condition requires interdisciplinary efforts and new technologies, that can potentially encompass and integrate the different dimensions of the glomerulus and the complexity of the diabetic milieu. These efforts will lead us to new insights into the pathogenesis of DKD and the discovery of novel therapeutic targets.

## Author Contributions

ID drafted, prepared, and edited the manuscript.

## Conflict of Interest Statement

The author declares that the research was conducted in the absence of any commercial or financial relationships that could be construed as a potential conflict of interest.
