# Corrigendum: Glomerular Endothelial Cell Stress and Cross-Talk With Podocytes in Early Diabetic Kidney Disease

**DOI:** 10.3389/fmed.2018.00113

**Published:** 2018-04-26

**Authors:** Ilse Sofia Daehn

**Affiliations:** Division of Nephrology, Department of Medicine, Icahn School of Medicine at Mount Sinai, The Charles Bronfman Institute for Personalized Medicine, New York City, NY, United States

**Keywords:** diabetic kidney disease, glomerulus, crosstalk, podocyte, endothelial cell, ROS

There was a mistake in the Title. The correct version of the Title is: “Glomerular Endothelial Cell Stress and Cross-Talk With Podocytes in Early Diabetic Kidney Disease” instead of “Glomerular Endothelial Cells Stress and Cross-Talk With Podocytes in the Development of Diabetic Kidney Disease”. The author apologizes for the mistake. This error does not change the scientific conclusions of the article in any way.

The original article has been updated.

## Conflict of Interest Statement

The authors declare that the research was conducted in the absence of any commercial or financial relationships that could be construed as a potential conflict of interest.

